# Exacerbation of Hereditary Pyruvate Kinase Deficiency Following a Vegetable Juicing Fad Diet: A Case of Asymptomatic Jaundice Due to Hemolytic Anemia

**DOI:** 10.7759/cureus.83590

**Published:** 2025-05-06

**Authors:** Steven J Laxton

**Affiliations:** 1 Department of Emergency Medicine, The University of Tennessee Health Science Center (UTHSC) Nashville - Ascension Saint Thomas Rutherford, Murfreesboro, USA

**Keywords:** anemia, hemolytic anemia, hyperbilirubinemia, jaundice, pyruvate kinase, pyruvate kinase deficiency

## Abstract

Pyruvate kinase (PK) deficiency is a hereditary genetic disorder caused by an abnormal function of the enzyme pyruvate kinase. The enzyme function can range from near normal function to complete dysfunction of the enzyme PK. Just like the variability of enzyme function, this chronic illness can vary from mild anemia never needing intervention to severe anemia, multiorgan dysfunction, and death depending on the level of function of the enzyme PK.

This is a case report of a middle-aged female patient who presented to the emergency department for persistent jaundice over the past two months. The patient was found to have unconjugated hyperbilirubinemia secondary to hemolytic anemia on laboratory examination. This is secondary to a major change in diet. The patient changed to a “fad” diet of “vegetable juicing”, causing hemolytic anemia secondary to exacerbation of hereditary PK deficiency. This case highlights an abnormal presentation of a common complaint, jaundice, from the perspective of the emergency department in the setting of known hereditary enzyme deficiency and major diet change.

No emergency interventions were required in the emergency department and the patient was able to be discharged home with gastroenterology follow-up and was instructed to return to prior heterogenous and balanced diet, which resulted in a complete resolution of symptoms.

This case report presents a common chief complaint in an emergency department, caused by a rare disorder, exacerbated by an unlikely and previously unreported cause (vegetable juicing diet) based on current literature review.

## Introduction

When a patient has a presenting symptom of jaundice, it can be secondary to multiple etiologies. There are three broad categories of hyperbilirubinemia that can aid in making the diagnosis of jaundice: (i) cholestatic (i.e. choledocholithiasis), (ii) isolated (i.e. red blood cell hemolysis), or (iii) in combination with hepatocellular injury (i.e. acetaminophen overdose). Typically, cholestatic will be associated with pain secondary to swelling of the liver capsule. Isolated will be variable in presentation as it can be secondary to multiple etiologies [1].

As always, history will take precedence [1] for aiding in diagnostic and treatment efficiency as someone with a recent acetaminophen overdose would change evaluation, management, and disposition. When evaluating a patient with hyperbilirubinemia, it is also key to focus on clues that may lead to the etiology of a patient's hyperbilirubinemia such as protracted or recent large volume consumption of alcohol, medication use and dosage, history of abdominal surgery (i.e. recent gallbladder operation), recent medication overdose, and history of hereditary diseases [2].

The physical exam is also of vital importance as this can give key clues to the etiology of the patient’s illness. Important findings may include right upper quadrant abdominal pain, findings consistent with liver disease such as ascites, splenomegaly, spider angiomas, varices near the umbilicus, and gynecomastia [2].

Physical exam to determine the most likely cause is the mainstay of evaluation followed by lab analysis. This is important to help differentiate etiology. The suggested laboratory exam includes complete blood count, metabolic profile with renal function, hepatic function, and serum total and indirect (unconjugated) bilirubin.

This patient was found to have isolated jaundice. Laboratory exam showed elevated lactate dehydrogenase (LDH) and low haptoglobin levels, indicating that this was likely secondary to hemolytic anemia. This patient has a known enzyme deficiency of pyruvate kinase deficiency but has never required transfusion or needed further treatment other than intermittent monitoring of hemoglobin and hematocrit.

Fad diets come and go and always leave a string of injured followers. Such examples can be seen in recent popular and mainstream “keto diet” [3] or simply carbohydrate restriction diet [4]. This diet is without exception and patients with genetic predisposition could be at an elevated risk of an adverse outcome.

The fad diet that this patient chose was a trending diet of “vegetable juicing” [5,6]. This diet is variable among participants with regard to which types of vegetables they decide to juice and ingest. In this case, the patient chose to exclusively eat cucumbers, spinach, and celery that she would then blend with water and make into a smoothie to drink. 

Pyruvate kinase deficiency (PKD) is a hereditary disorder of an enzyme within the glycolytic pathway causing decreased or low activity. The gene that codes for pyruvate kinase is an autosomal gene that is inherited in a recessive fashion. The estimated prevalence of PKD ranges from 3.2 to 8.5 cases per million of the Western population [7]. PKD can present in a variable fashion from no symptoms to mild anemia or severe anemia and multiorgan dysfunction depending on gene penetrance and enzyme activity. When presented as a homozygous inheritance, this diagnosis is usually made in the newborn period due to requirement of phototherapy and persistent jaundice [8]. However, when presented as a heterozygous inheritance, the diagnosis is usually delayed due to the spectrum of enzyme activity causing anemia [9] to anywhere from severe anemia requiring frequent transfusions to mild or borderline normal anemia.

Hemolysis may occur in stressful conditions, which in this case was precipitated by caloric restriction, decreased protein intake, or a combination of decreased calorie and protein ingestion in this case as the patient had no caloric-dense food intake such as bean- or legume-type food.

This presentation of asymptomatic unconjugated hyperbilirubinemia was initially confusing as the patient’s hereditary deficiency is a rare illness and, from the standpoint of prevalence, the likelihood of PKD being the root cause of jaundice was statistically low with many other diagnoses more likely.

This case report presents a common chief complaint received at the emergency department but caused by a rare disorder exacerbated by an uncommon and unreported cause (vegetable juicing diet) based on current literature review.

## Case presentation

A 38-year-old female patient presented to the emergency department following referral from primary care for asymptomatic jaundice. This patient has a past medical history of schizoaffective disorder and pyruvate kinase deficiency causing mild intermittent anemia since birth without ever needing a blood transfusion or additional therapy aside from intermittent laboratory monitoring. 

The history of present illness was only pertinent for a recent, one month prior, major diet change to a fad diet of “vegetable juicing” where one eats exclusive vegetables that are blended and liquefied, and the patient ingested mostly celery, cucumbers, and spinach in liquid form. There was a complete abstinence from meats, grains, and milk products. The patient said she had been participating in this diet for about three months and noticed a gradual worsening of jaundice over the past two months. She did not consume alcohol or did not have a history of liver disease or cirrhosis. She also denied any use of medications, including over-the-counter medications. 

The physical exam aside from jaundice and scleral icterus was unremarkable. The patient had no neurological findings, heart and lungs were unremarkable, and abdomen was non-distended, nontender and without findings of caput medusae, hepatomegaly, or hepatojugular reflux. The patient also had no lower extremity swelling. Skin was found with jaundice and without spider angiomas or petechial lesions. 

Laboratory examination was performed for complete blood count (CBC), comprehensive metabolic panel (CMP), direct bilirubin, LDH, haptoglobin, and reticulocyte count. The CBC was significant for a decreased hemoglobin and hematocrit of 10.9 gm/dL and 34%, respectively. The metabolic panel was noted to have an elevation in total bilirubin of 7.0 mg/dL. The direct bilirubin was only mildly elevated to 0.8 mg/dL, indicating the bilirubin elevation was due to indirect bilirubin. LDH was high at 295 U/L. The haptoglobin was decreased to 10 mg/dL (normal 40-200 mg/dL). The reticulocyte count was also elevated to 6%. See Table 1 for a complete list of laboratory values obtained and reference ranges. 

**Table 1:** Laboratory Findings Table of laboratory findings for basic metabolic panel (BMP), complete blood count (CBC), and miscellaneous tests with corresponding reference ranges. BUN: blood urea nitrogen; eGFR: estimated glomerular filtration rate; T. protein: total protein; Alk Phos: alkaline phosphatase; AST: aspartate transaminase; ALT: alanine aminotransferase; AGAP: anion gap; WBC: white blood cells; RBC: red blood cells; Hgb: hemoglobin; HCT: hematocrit; MCV: mean corpuscular volume; MCH: mean corpuscular hemoglobin; MCHC: mean corpuscular hemoglobin concentration; RDW: red cell distribution width

Test	Result (*if abnormal)	Normal Range
Basic Metabolic Panel		
Na	137 mmol/L	135-145 mmol/L
K	3.5 mmol/L	98-106 mmol/L
Cl	101 mmol/L	98-107 mmol/L
CO_2_	21 mmol/L*	23-29 mmol/L
BUN	4 mg/dL*	7-20 mg/dL
Creatinine	0.6 mg/dL	0.6-1.2 mg/dL
eGFR	120 mL/min*	>60 mL/min/1.73 m²
Glucose	71 mg/dL	70-99 mg/dL
Calcium	9.8 mg/dL	8.5-10.5 mg/dL
T. Protein	7.6 gm/dL	6.0-8.3 g/dL
Albumin	5.1 gm/dL	3.5-5.0 g/dL
Bilirubin total	7.0 mg/dL*	0.1-1.2 mg/dL
Alk phos	41.0 U/L	44-121 IU/L
AST	18 U/L	10-40 IU/L
ALT	15 U/L	7-56 IU/L
AGAP	15 mEq/L	8-16 mEq/L
Bilirubin direct	0.8 mg/dL*	0.0-0.3 mg/dL
Ancillary tests		
Lipase	39.0 U/L	10-140 U/L
Pregnancy	Negative	<5 mIU/mL (non-pregnant)
LDH	285.0 U/L*	140-280 U/L
Haptoglobin	10 mg/dL*	30-200 mg/dL
Complete blood count		
WBC	5.1 x10x3/mm^3^	4.0-11.0 x 10³/µL
RBC	3.36 x10^6/mm^3^ L	4.2-5.4 x 10⁶/µL
Hgb	10.9 gm/dL*	12.1-15.1 g/dL
HCT	34.5% L	36.1-44.3%
MCV	102.7 fL*	80-100 fL
MCH	32.4 pg	27-33 pg/cell
MCHC	31.6 gm/dL *	32-36 g/dL
RDW	12.00%	11.5-14.5%
Platelet	172 x10^3^/mm^3^	150-450 x 10³/µL
Reticulocyte %	6%*	0.5-2.5%

Gastroenterology was consulted from the emergency department and the specialist recommended that the patient have variability in diet and include grains and meat in her diet and follow-up as an outpatient. The outpatient course was also insignificant, and the patient returned to the emergency department six weeks (about one and a half months) later with a complete resolution of jaundice after switching back to her diet prior to “vegetable juicing.”

## Discussion

Jaundice is a relatively common presenting symptom among adults in the emergency department. This, however, is usually cholestatic (i.e., choledocholithiasis) or in combination with hepatocellular injury and infrequently due to isolated causes such as hemolytic anemia [10] or, less likely, exacerbation of genetic disease as in this case. 

When evaluating hyperbilirubinemia, the first question that should be posed is whether the bilirubin is conjugated or unconjugated. If it is conjugated hyperbilirubinemia, then it is typically secondary to liver causes and can be further classified as intrahepatic or extrahepatic hyperbilirubinemia. When it is unconjugated, then it can be further classified into categories of increased bilirubin production (i.e., red blood cell hemolysis), decreased uptake of bilirubin by the liver (i.e., congestive hepatopathy (CHF)), or decreased conjugation (i.e., Crigler Najar/Gilbert disease). In this case, the patient has a predisposition for red blood cell hemolysis due to pyruvate kinase deficiency. Laboratory examination confirmed the suspicion of hemolysis with elevated LDH and decreased haptoglobin. There was also no sign of liver injury as the patient had normal albumin levels and no elevation of liver enzyme levels. 

Fortunately, for this patient, the anemia did not require transfusion, which is a common outcome in exacerbations of pyruvate kinase deficiency [11] and only diet change was required to increase caloric intake as well as have a well-balanced diet for nutritional support.

As of March 10, 2025, there is no prior report of an exacerbation of pyruvate kinase deficiency causing hemolytic anemia secondary to major diet change when PubMed or NIH was searched to review current medical literature. 

As “fad” diets are becoming increasingly more common secondary to an increase in obesity [12], it is likely that physicians will continue to encounter complications of fad diets [13-15] such as in this report and many other prior reports.

## Conclusions

Jaundice is a relatively common presenting symptom that is encountered in the emergency department. It is usually readily detected and appropriately diagnosed as there is usually an easily identifiable etiology based on history and physical examination. In the case presented, clinical suspicion arose as the cause of jaundice was isolated and proper streamlined testing was obtained to arrive at the diagnosis. 

The evaluation of jaundice and hyperbilirubinemia relies extensively on laboratory examination findings and there are many established algorithms that will aid in making the diagnosis. Like in most cases, history was key to making the diagnosis and establishing a treatment plan for this patient. In this case, there were no emergency interventions needed; however, in many cases there can be critical illness. After following a well-balanced diet, jaundice completely resolved in the patient.
